# Genome analysis and CRISPR typing of *Salmonella enterica* serovar Virchow

**DOI:** 10.1186/1471-2164-15-389

**Published:** 2014-05-21

**Authors:** Nathan L Bachmann, Nicola K Petty, Nouri L Ben Zakour, Jan M Szubert, John Savill, Scott A Beatson

**Affiliations:** Australian Infectious Diseases Research Centre and School of Chemistry and Molecular Biosciences, The University of Queensland, Brisbane, QLD Australia; The ithree institute, University of Technology Sydney, Sydney, NSW Australia; Public Health Microbiology Laboratory, Public and Environmental Health, Forensic and Scientific Services, Queensland Health, Brisbane, QLD Australia

## Abstract

**Background:**

*Salmonella enterica* subsp*. enterica* serovar Virchow has been recognized as a significant health burden in Asia, Australia and Europe. In addition to its global distribution, *S.* Virchow is clinically significant due to the frequency at which it causes invasive infections and its association with outbreaks arising from food-borne transmission. Here, we examine the genome of an invasive isolate of *S.* Virchow SVQ1 (phage type 8) from an outbreak in southeast Queensland, Australia. In addition to identifying new potential genotyping targets that could be used for discriminating between *S.* Virchow strains in outbreak scenarios, we also aimed to carry out a comprehensive comparative analysis of the *S.* Virchow genomes.

**Results:**

Genome comparisons between *S.* Virchow SVQ1 and *S.* Virchow SL491, a previously published strain, identified a high degree of genomic similarity between the two strains with fewer than 200 single nucleotide differences. Clustered Regularly Interspaced Palindromic Repeats (CRISPR) regions were identified as a highly variable region that could be used to discriminate between *S.* Virchow isolates. We amplified and sequenced the CRISPR regions of fifteen *S.* Virchow isolates collected from seven different outbreaks across Australia. We observed three allelic types of the CRISPR region from these isolates based on the presence/absence of the spacers and were able to discriminate *S.* Virchow phage type 8 isolates originating from different outbreaks. A comparison with 27 published *Salmonella* genomes found that the *S.* Virchow SVQ1 genome encodes 11 previously described *Salmonella* Pathogenicity Islands (SPI), as well as additional genomic islands including a remnant integrative conjugative element that is distinct from SPI-7. In addition, the *S.* Virchow genome possesses a novel prophage that encodes the Type III secretion system effector protein SopE, a key *Salmonella* virulence factor. The prophage shares very little similarity to the SopE prophages found in other *Salmonella* serovars suggesting an independent acquisition of *sopE*.

**Conclusions:**

The availability of this genome will serve as a genome template and facilitate further studies on understanding the virulence and global distribution of the *S.* Virchow serovar, as well as the development of genotyping methods for outbreak investigations.

**Electronic supplementary material:**

The online version of this article (doi:10.1186/1471-2164-15-389) contains supplementary material, which is available to authorized users.

## Background

*Salmonella enterica* subsp*. enterica* serovar Virchow is commonly associated with gastroenteritis, but it is also known to cause invasive systemic infections [1–4]. Outbreaks of serovar Virchow are a significant public health risk in many European, Asian and Oceanic countries [3, 5–8]. Currently, *S.* Virchow is one of most prevalent *Salmonella* serovars in Australia and outbreaks can occur through food-borne transmission via contaminated fruit and vegetables and poor food handling practices [2, 9, 10]. In order to track outbreaks of *S.* Virchow, molecular subtyping methods are needed to discriminate between strains, however, no such typing scheme currently exits.

Phage typing is a well-established method for discriminating between *Salmonella* strains based on their susceptibility to lytic infection by specific bacteriophages [11, 12]. However, discrepancies in phage typing results between different laboratories have been reported [13]. There are also several nucleic acid-based typing methods, including pulsed-field gel electrophoresis (PFGE), which involves using restriction enzymes to cut bacterial DNA into fragments and analysing the banding patterns following gel electrophoresis [14, 15]. However, PFGE has limitations in reproducibility and the results can be ambiguous, and is also limited in its ability to discriminate between different strains [16]. On the other hand, multiple-loci variable-number tandem repeat analysis (MLVA), a PCR based method used to detect variation in the number of repeat units in tandem repeat sequences [17, 18], provides improved level of discrimination for many *Salmonella* serovars compared to PFGE [19]. In addition, Multi Locus Sequence Typing (MLST) also allows greater discrimination between serovars. It involves detecting allelic differences in the sequences of various housekeeping genes [20–22] and can also been extended to include virulence genes [23]. Even greater resolution can be achieved by identifying single nucleotide polymorphisms (SNPs) as genotyping targets from whole genome sequence (WGS) data, with schemes available for serovars like *S.* Typhimurium [24, 25].

Whilst there are MLVA and SNP typing schemes available for many *Salmonella* serovars there are currently none available for discriminating between the different *S.* Virchow phage type (PT) strains [26–30]. By MLST, *S.* Virchow strains belong to the eBurst Group BG9, however, the majority of BG9 strains in the MLST database are classified as sequence type 16 (ST16) [31]. Therefore, additional genotyping targets with a greater degree of discrimination between strains are required for subtyping *S.* Virchow.

Clustered regularly interspaced short palindromic repeats (CRISPRs) have recently been used to subtype more than 9 major *Salmonella* serovars including Typhimurium, Newport and Enteritidis [32–34]. CRISPRs are sequences consisting of 21–47 nucleotides that are repeated in tandem separated by non-repetitive sequences of a similar size [35]. A cluster of genes known as CRISPR-associated genes (*cas*) is often found near the start of CRISPR regions [36]. CRISPRs function as a defense system against foreign DNA such as plasmids and bacteriophage by producing small RNA (sRNA) that can silence foreign mRNA, similar to a RNA interference system [37]. Generally, CRISPRs vary between *Salmonella* strains in the number of repeats/spacers [32, 33, 38]. Prophages are also useful for genotyping *Salmonella* but have not as yet been applied to *S.* Virchow as a routine epidemiological tool [39, 40].

Like the majority of bacteria, mobile genetic elements such as plasmids, bacteriophages and insertion sequence elements are the main drivers of gene flux in *Salmonella* [41–44]. This organism has acquired many of its virulence genes from mobile elements and they are often found within islands, referred to as *Salmonella* Pathogenicity Islands (SPIs) [45]. Other virulence factors such as Type III secreted effectors can be found encoded in prophage regions [42, 46]. Until recently only one other *S.* Virchow genome had been reported [38]. The genome for *S.* Virchow SL491, a PT25 strain that was isolated in the United States, was studied as part of a broader comparative study of 28 *S.* enterica strains [38]. Similarly, during the preparation of this manuscript, a second *S.* Virchow draft genome was reported as part of a large WGS phylogenetic analysis of 78 Salmonella serovars. These studies showed that *S.* Virchow strains were most closely related to strains of the Heidelberg serovar and carry distinctive CRISPR regions [38, 47], however, a comprehensive genomic comparison of different *S.* Virchow strains has yet to be reported.

Here we report our comparative analyses of the genome of an Australian isolate of *S.* Virchow PT 8 (SVQ1) with the published genome of *S.* Virchow PT25 (SL491). We report a comparative analysis with 27 other *Salmonella* genomes that reveals the mobile element content of *S.* Virchow strains and furthers our understanding of the evolution of this important food-borne pathogen. We have also identified new discriminatory genotyping targets that can be combined with existing *Salmonella* genotyping schemes to elucidate the relatedness of individual *S.* Virchow isolates.

## Results

### Whole genome comparison of *S.* Virchow SVQ1 and *S.* Virchow SL491

**Figure 1:**
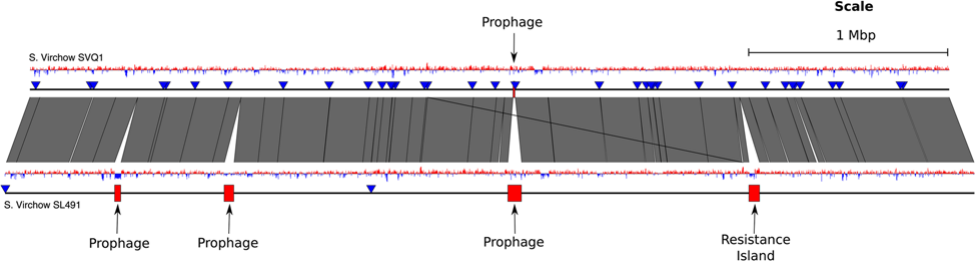
**Comparison between the draft genomes of** ***Salmonella*** **Virchow SVQ1 and** ***Salmonella*** **Virchow SL491.** Regions of differences are shown as red boxes and labeled accordingly. Vertical blocks between the genomes represents regions of shared similarity according to BLASTn (Nucleotide identity >99%) [49]. The GC content for both genomes is shown as graphs, red indicates above average GC content while blue indicates below average GC content. Contig boundaries are marked with blue triangles. The resistance island carries the *rmtC* gene, which confers resistance to aminoglycosides [38]. The image was prepared using EasyFig [50].

The draft genome of *S.* Virchow SVQ1 (PT8) consists of a 4.67 Mbp chromosome and four plasmids that range from 2.5 to 37 kb (Additional file 1: Table S1). Differences between the *S.* Virchow SVQ1 chromosome and *S.* Virchow SL491 chromosome include 13 genes that makes up a remnant prophage in SVQ1. The *S*. Virchow SL491 genome is larger than S. Virchow SVQ1 genome with addition of 280 genes that are distributed amongst three prophage and a genomic island that were likely acquired via lateral gene transfer (LGT) (Figure 1). Read mapping was used to confirm that the observed absence of *S.* Virchow SL491 prophage and islands regions in *S.* Virchow SVQ1 was genuine and not as the result of assembly errors (data not shown). The genomic island encodes the aminoglycoside resistance gene *rmtC* and a partial mercury resistance transposon operon [38]. *S.* Virchow SVQ1 carries four plasmids that are absent in *S.* Virchow SL491. The largest SVQ1 plasmid shares 96–98% identity across 78% of the non-virulence plasmid pOU1114 found *S.* Dublin and encodes a conjugative transfer system [48]. The other three plasmids are non-conjugative and are each unambiguously assembled into a single circular contig (Additional file 1: Table S1). We detected 195 variants within coding regions between the two *S.* Virchow genomes, including 166 SNPs, 13 single nucleotide frame-shift indels, and 5 three-nucleotide in-frame indels (Additional file 2: Table S2). By comparison, the genome of *S.* Heidelberg SL497 differs from the genome of *S.* Virchow SVQ1 by approximately 34,000 SNPs.

### SNP containing genes provide limited discrimination of S. Virchow isolates

We tested 11 genes that contained SNPs between the genomes of *S.* Virchow SVQ1 and *S.* Virchow SL491 that may be potential discriminatory genotyping targets (Additional file 3: Table S3). Amplicons were sequenced from 45 *S.* Virchow isolates that had been collected from various outbreaks in Australia (Table 1), as well as *S.* Virchow SVQ1 and *S.* Virchow SL491. Only one gene out of 11, encoding a probable pyruvate-flavodoxin oxidoreductase (locus tag: Sesv_1374), was determined to be a potential target for discriminating Australian *S.* Virchow PT8 isolates. In *S.* Virchow SVQ1, and four other related isolates from same outbreak, this gene contained a Cytosine (C) at position 1428 in the 3.5 kb gene, whereas the remaining isolates (including several PT8 strains from other outbreaks) had a Thymine (T) in this position. The remaining 10 genes were found to have a conserved sequence in all 45 Australian *S.* Virchow isolates. In all cases the sequencing of SVQ1 and SL491 genotyping candidates was consistent with the original SNP prediction.

**Table 1:** **List of** ***S.*** **Virchow isolates that were used in this study**

Num of strains	Phage type	Cluster (C) or outbreak (O)^1^	Source: faecal (F) or blood (B)^2^	Origin^3^	Year	Reference^4^
1	PT8	C	F	QLD	2008	This study
5	PT8	O	4 F, 1B	QLD	2007	This study
1	PT25	U	F	USA	2005	[38]
9	PT8	C	F	NT	2006	NEPSS, 2006, p11
9	PT8	C	F	WA	2005	NEPSS, 2005, p13
5	PT8	C	F	QLD	2008	This study
3	PT8	C	F	QLD	2004	NEPSS, 2005, p9
7	PT17	C	4 F, 3B	QLD	2001	NEPSS 2001
6	PT34	O	F	VIC	2001	NEPSS, 2001
						SEPT2002, p13
1	PT25	C	F	QLD	2005	NEPPS annual report 2005, 2006 1/06, p12

^1^A cluster (C) is a group of cases that occurred in a specific place and time. An outbreak (O) is an incident of cases where the source of the infection is known. The University of Calgary (U) provided this isolate.

^2^ F, Faecal isolate; B, Blood isolate.

^3^QLD, Queensland; NT, Northern Territory; WA, Western Australia; VIC, Victoria.

^4^National Enteric Pathogens Surveillance Scheme. Annual Reports 2001–2008. Melbourne: Microbiological Diagnostic Unit, University of Melbourne.

### CRISPRs as potential targets for discrimination of *S.* Virchow isolates

**Figure 2:**
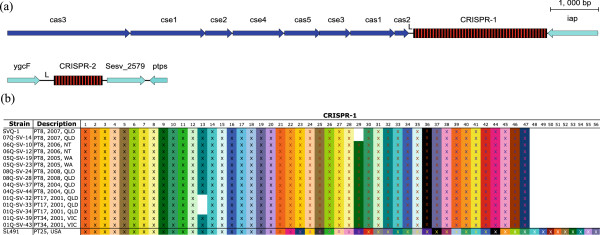
**Comparison of** ***S.*** **Virchow CRISPR regions. a.** Schematic representation of the two CRISPR regions in *S.* Virchow SVQ1. Direct repeats are shown as black rectangles and the spacers are shown as red rectangles. CRISPR-associated genes (*cas*) and other flanking genes are represented by dark-blue and light-blue arrows, respectively. L stands for the leader sequence. The genes that are flanking CRISPR-1 are associated with the locus tags Sesv_2565 to Sesv_2573 and the genes flanking CRISPR-2 are associated with the locus tags Sesv_2578 to Sesv_2580, respectively. This image was prepared using Easyfig [50]. **b.** Representation of spacer arrangement in CRISPR-1 in 15 Australian *S.* Virchow isolates. Each unique spacer is represented by a combination of background colour and the colour of the X character. White gaps represent the absence of a particular spacer. Strains are grouped by phage type, the year it was collected and location. The spacer arrangement of CRISPR-1 from the genome of *S.* Virchow SL491 is also shown. QLD = Queensland, NT = Northern Territory, WA = Western Australia and VIC = Victoria.

Like the majority of *Salmonella* serovars, *S.* Virchow SVQ1 has two CRISPRs: CRISPR-1, which is 2.7 kb in length and has 45 spacers, and CRISPR-2, which is 1 kb in length and has 16 spacers (Figure 2a). Comparisons of CRISPRs in *S.* Virchow SVQ1 and *S.* Virchow SL491 revealed that CRISPR-1 is substantially larger in SL491 with 55 spacers. However, only the first 21 spacers are conserved between both *S.* Virchow genomes, indicating that there may be sufficient variability within this region to sub-type *S.* Virchow strains. CRISPR-2 is identical between the two *S.* Virchow genomes.

The CRISPR-1 region was sequenced in fifteen Australian *S.* Virchow isolates consisting of various phage types (PT8, PT17 and PT34) to determine the level of variation between strains (Table 2). The fifteen strains selected include at least two strains from each of seven different outbreaks, that have occurred between 2001 and 2008. Three allelic types of CRISPR-1 were observed based on the presence/absence of particular spacer sequences (Figure 2b). CRISPR typing was able to distinguish *S.* Virchow SVQ1 (PT8) and a second PT8 isolate from the same outbreak (07Q-SV-14) from other Australian PT8 isolates due to the absence of spacer 29. The absence of this spacer distinguishes *S.* Virchow SVQ1 and 07Q-SV-14 from the other PT8 isolates, demonstrating that CRISPRs can be used to help discriminate between *S.* Virchow strains within a phage type. Notably, PT17 isolates are characterized by the absence of a different spacer (Figure 2b).

**Table 2:** **List of Australian** ***S.*** **Virchow isolates used in the CRISPR analysis**

Strain	Phage type	Year	State	Source^1^	Accession number
SVQ1	PT8	2007	Queensland	This study	[GenBank:AZMP01000000]
07-SV-14	PT8	2007	Queensland	This study	[GenBank:KF931136]
06-SV-10	PT8	2006	Northern Territory	NEPSS, 2006, p11	[GenBank:KF931134]
06Q-SV-12	PT8	2006	Northern Territory	NEPSS, 2006, p11	[GenBank:KF931135]
05Q-SV-19	PT8	2005	Western Australia	NEPSS, 2005, p13	[GenBank:KF931132]
05Q-SV-23	PT8	2005	Western Australia	NEPSS, 2005, p13	[GenBank:KF931133]
08Q-SV-24	PT8	2008	Queensland	This study	[GenBank:KF931137]
08Q-SV-28	PT8	2008	Queensland	This study	[GenBank:KF931138]
04Q-SV-37	PT8	2004	Queensland	NEPSS, 2005, p9	[GenBank:KF931130]
04Q-SV-44	PT8	2004	Queensland	NEPSS, 2005, p9	[GenBank:KF931131]
01Q-SV-32	PT17	2001	Queensland	This study	[GenBank:KF931125]
01Q-SV-33	PT17	2001	Queensland	This study	[GenBank:KF931126]
01Q-SV-34	PT17	2001	Queensland	This study	[GenBank:KF931127]
01Q-SV-39	PT34	2001	Victoria	NEPSS, 2001 SEPT2002, p13	[GenBank:KF931128]
01Q-SV-43	PT34	2001	Victoria	NEPSS, 2001 SEPT2002, p13	[GenBank:KF931129]

^1^National Enteric Pathogens Surveillance Scheme. Annual Reports 2001–2008. Melbourne: Microbiological Diagnostic Unit, University of Melbourne.

### Genomic analysis of *S.* Virchow SVQ1

**Figure 3:**
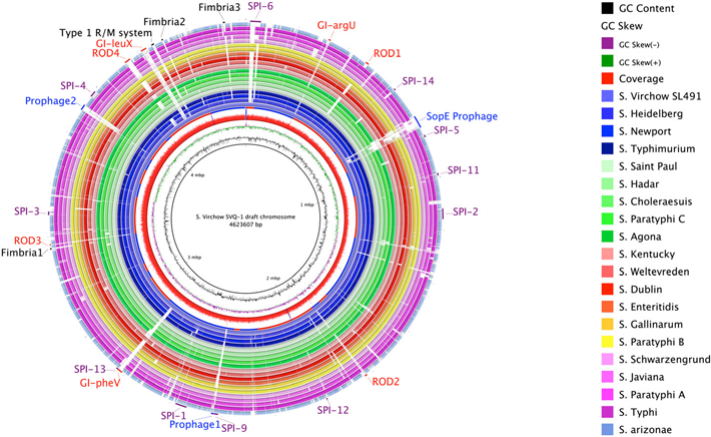
**Blast ring image of** ***S.*** **Virchow SVQ1 genome.** The innermost rings show *S*. Virchow SVQ1 genome position (mbp = Megabases), GC content (black) and GC skew (purple/green) and read coverage (red). The contig boundaries for the *S.* Virchow SVQ1 genome are shown as alternating red and blue bars on the fifth innermost ring. The remaining rings show BLASTn comparison of the 27 other *Salmonella* genomes listed in Table 3, against *S.* Virchow SVQ1 (in some cases multiple genomes are grouped into a single ring). BLASTn matches with an identity between 90% and 100% are coloured, while non-matching regions appear as blank spaces in each ring. The outer ring contains annotations, coloured according to function: regions variable in other *Salmonella* genomes such as fimbrial usher/chaperone operons and a Type I restriction-modification system (black); prophage regions (blue); genomic islands in recognised integration sites (GI-*argU*, GI-*pheV* and GI-*leuX*) and other regions of difference (ROD1-4) (red). Green labels refer to the *Salmonella* Pathogenicity Islands present in *S.* Virchow. The image was prepared using BRIG [59].

**Figure 4:**
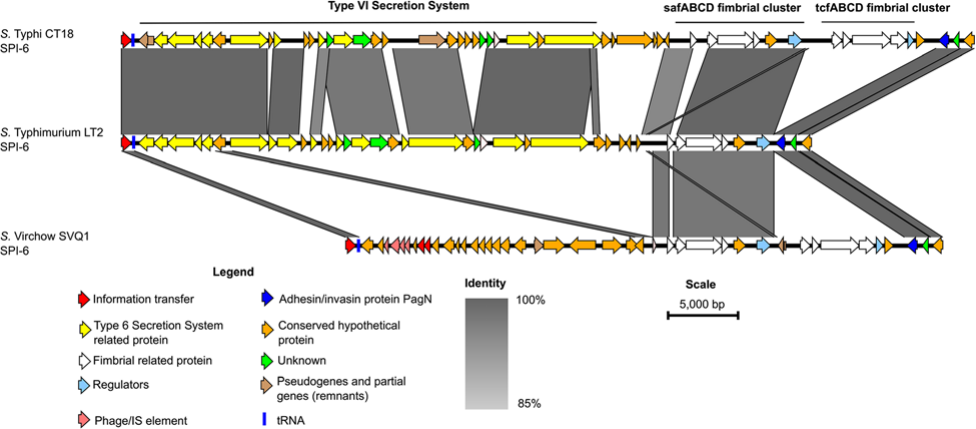
**Nucleotide comparison of SPI-6 from** ***S.*** **Typhi CT18,** ***S.*** **Typhimurium LT2 and** ***S.*** **Virchow SVQ1.** Grey vertical blocks indicate regions of shared similarity shaded according to BLASTn. The coloured arrows represent genes. The functions of the genes have been inferred from BLAST searches. The intact SPI-6 in S. Typhi CT18 carries a Type VI secretion system (T6SS) and two fimbrial clusters (*saf* and *tcf*) and encodes the adhesin/invasin protein PagN. In *S.* Typhimurium LT2 the *tcf* fimbrial cluster is absent. In *S.* Virchow the T6SS is missing but both fimbrial clusters are present. The image was prepared using Easyfig [50].

The genome of *S.* Virchow SVQ1 was compared with 27 *Salmonella* genomes to determine genetic differences between Virchow and the other serovars (Table 3). The comparison revealed that the genomic backbone of *S.* Virchow is similar to the genomes of other *Salmonella* serovars, including key virulence factors. The *S.* Virchow genome encodes the two Type III secretion systems that are conserved in all *Salmonella* serovars and are encoded on *Salmonella* Pathogenicity Islands (SPI-1 and SPI-2). *S.* Virchow also carries nine other known SPIs that are conserved within other *Salmonella* genomes, with the exception of SPI-6 (Figure 3 and Additional file 4: Table S4). The intact SPI-6 island in *S.* Typhi CT18 carries a Type VI Secretion System (T6SS), two fimbrial gene clusters (*saf*ABCD and *tcf*ABCD) and the invasin, PagN [51, 52]. However, the SPI-6 in the *S.* Virchow genome is missing the T6SS but it still possesses the two fimbrial clusters and *pagN* (Figure 4).

**Table 3:** **Genome sequences used in the genomic comparison**

Ring^1^	Genome	Strain	GenBank accession	Reference
6	*Salmonella enterica subsp. enterica* serovar Virchow	SL491	ABFH00000000	[38]
7	*Salmonella enterica subsp. enterica* serovar Heidelberg	SL476	CP001120	[38]
	*Salmonella enterica subsp. enteri*ca serovar Heidelberg	SL486	ABEL00000000	[38]
8	*Salmonella enterica subsp. enterica* serovar Newport	SL254	CP001113	[38]
	*Salmonella enterica subsp. enterica* serovar Newport	SL317	ABEW00000000	[38]
9	*Salmonella enterica subsp. enterica* serovar Typhimurium	LT2	AE006468	[53]
	*Salmonella enterica subsp. enterica* serovar Typhimurium	UK-1	CP002614	[54]
10	*Salmonella enterica subsp. enterica* serovar Saintpaul	SARA23	ABAM02000001	[38]
	*Salmonella enterica subsp. enterica* serovar Saintpaul	SARA29	ABAN00000000	[38]
11	*Salmonella enterica subsp. enterica* serovar Hadar	RI_05P066	ABFG01000000	[38]
12	*Salmonella enterica subsp. enterica* serovar Choleraesuis	SC-B67	AE017220	[55]
13	*Salmonella enterica subsp. enterica* serovar Paratyphi C	RKS4594	CP000857	[56]
14	*Salmonella enterica subsp. enterica* serovar Agona	SL483	CP001138	[38]
15	*Salmonella enterica subsp. enterica* serovar Kentucky	CDC 191	ABEI01000000	[38]
	*Salmonella enterica subsp. enterica* serovar Kentucky	SL475	ABAK02000001	[38]
16	*Salmonella enterica subsp. enterica* serovar Weltevreden	HI_N05-537	ABFF00000000	[38]
17	*Salmonella enterica subsp. enterica* serovar Dublin	CT_02021853	CP001144	[38]
18	*Salmonella enterica subsp. enterica* serovar Enteritidis	P125109	AM933172	[57]
19	*Salmonella enterica subsp. enterica* serovar Gallinarum	287/91	AM933173	[57]
20	*Salmonella enterica subsp. enterica* serovar Paratyphi B	SPB7	CP000886	W.U. Genome Sequencing Centre
21	*Salmonella enterica subsp. enterica* serovar Schwarzengrund	SL480	ABEJ01000000	[38]
	*Salmonella enterica subsp. enterica* serovar Schwarzengrund	CVM19633	CP001127	[38]
22	*Salmonella enterica subsp. enterica* serovar Javiana	SL478	ABEH00000000	[38]
23	*Salmonella enterica subsp. enterica* serovar Paratyphi A	ATCC9150	CP000026	[53]
24	*Salmonella enterica subsp. enterica* serovar Typhi	CT18	AL513382	[41]
	*Salmonella enterica subsp. enterica* serovar Typhi	Ty2	AE014613	[58]
25	*Salmonella enterica subsp. arizonae* serovar 62:z4,z23	RKS2980	CP000880	W.U. Genome Sequencing Centre

^1^Genomes are listed as they appear in Figure 3, from innermost to outermost. Rings 1 to 5 correspond to *S*. Virchow SVQ1 genome position, GC skew, GC content, coverage and contig boundaries, respectively.

The whole genome comparison with other *Salmonella* serovars revealed that *S.* Virchow SVQ1 contains several regions of difference (RODs) (Additional file 5: Table S5). RODs represent sequences that are present in *S.* Virchow SVQ1 but absent in most other *Salmonella* genomes. These RODs include three putative genomic islands, defined as RODs that contained integrase genes or evidence of integrase mediated insertion (i.e. flanking direct repeats) in chromosomal integration hot-spots (GI-*argU*, GI-*pheV*, and GI-*leuX*), and three putative prophage elements (Figure 3 and Additional file 5: Table S5). Putative prophage elements could be distinguished from genomic islands by the presence phage structural or replication genes. Flanking direct repeats could be defined at the boundaries of the three prophage regions and GI-*argU* (Additional file 5: Table S5). There are also several other RODs including the 9.4 kb O-antigen biosynthetic gene cluster between *gln* and *galF* (ROD2), as well as three chaperone-usher fimbrial clusters that are sporadically distributed amongst other *Salmonella* serovars (Additional file 5: Table S5). *S.* Virchow also encodes the R-, M- and S- subunits characteristic of a Type I restriction modification system: Sesv_4171, Sev_4170, Sesv_4169 each exhibit 91%, 97% and 52% amino acid identity with the corresponding subunits of the EcoAI enzyme (http://rebase.neb.com/rebase/enz/EcoAI.html), respectively. The DNA-recognition domain (pfam: Methylase_S) of the S-subunit is unique to *S.* Virchow suggesting that the *S.* Virchow heteromeric enzyme may resemble EcoAI mechanistically, but may have different sequence specificity.

GI-*pheV* is a 19.6 kb genomic island that is only found in two other *Salmonella* genomes (Figure 3) and has likely been acquired by LGT followed by integration into tRNA^pheV^. Notably it carries an orphan cytosine C5-methyltransferase (Sesv_2771) that may play a role in global regulation by site-specific DNA methylation throughout the genome. GI-*pheV* is inserted directly adjacent to SPI-13, which is a 7.4 kb pathogenicity island encoding a putative lyase, a hydrolase, an oxidase, and an arylsulphatase regulator and is known to be involved in systemic infection of mice and replication inside murine macrophages [60, 61]. All 47 *S.* Virchow isolates in this study were found to contain GI-*pheV* on the basis of PCR amplification across the 5′ and 3′ boundaries of the island (Additional file 6: Table S6).

GI-*leuX* is a 22.2 kb region inserted next to tRNA^leuX^ in place of the SPI-10 which is found in *S.* Typhi CT18 and *S.* Enteritidis P125109 (Figure 3). The island encodes an integrase and a degraded genomic island type IV secretion system (GI-T4SS), indicating that it appears to be a remnant integrative conjugative element (ICE) ([62]). Although the majority of conjugal transfer genes are missing, the *S.* Virchow GI-*leuX* encodes the archetypal GI-T4SS conjugative coupling protein *traD/virD4* gene (locus tag: *sesv_4134*), albeit with a frame-shift that truncates VirD4 by 53 amino acids*.* When compared with previously defined representative T4SS sequences [62], *S.* Virchow VirD4 shares the most similarity (57-60% amino acid identity) with VirD4 homologs from the related and previously characterized ICEs *S.* Typhi CT18 SPI-7 (locus tag: Sty_4562) [63], *H. influenzae* ICE*Hin*1056 (locus tag: p1056.35) [64] and *P. aeruginosa* PAP-I (locus tag: RL047) [65]. Interestingly, the degraded GI-T4SS region encoded in GI-*leuX* shares ~90% nucleotide identity with GI-T4SS regions within the complete genomes of *Klebsiella pneumoniae* strains 1084 [GenBank:CP003785] and NTUH-K2044 [GenBank:AP006725], suggesting that GI-*leuX* belongs to a larger sub-group of uncharacterized ICEs. Like GI-*pheV*, a GI-*leuX* was identified in all 47 *S.* Virchow isolates in this study using PCR (Additional file 6: Table S6); however, further whole-genome sequencing would be required to determine the variability of this region amongst other strains of *S.* Virchow.

### *S.* Virchow SVQ1 carries a SopE prophage

**Figure 5:**
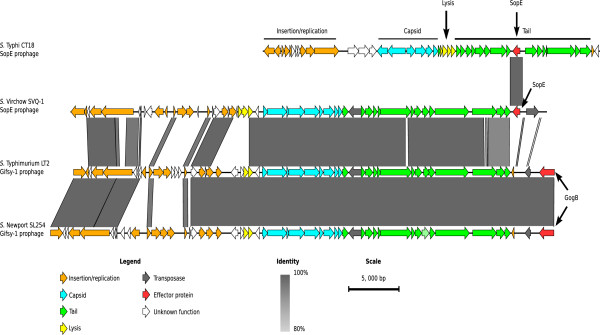
**Visual representation of the** ***S.*** **Virchow SVQ1 SopE prophage compared to other prophages.** Nucleotide comparison of the SopE prophages from *S.* Typhi CT18, and *S.* Virchow SVQ1 and the Gifsy-1 prophages from *S.* Typhimurium LT2 and *S.* Newport SL254, respectively. Grey vertical blocks indicate regions of shared similarity shaded according to BLASTn identity. The coloured arrows represent genes. The genes are coloured according to their predicted general functions, which has been inferred from BLAST searches, and are illustrated in the legend. The *sopE* gene and its conserved flanking sequence, which is called the *sopE* cassette, is 1.2 kb in length. The percentage identity between the *sopE* cassette of *S.* Typhi and *S.* Virchow is 97%. The image was prepared using Easyfig [50].

*S.* Virchow SVQ1 has three prophage regions encoded on the chromosome, only one of which is intact (Figure 3). Prophage 1 and 2 are incomplete ~8.9 kb and ~21 kb phage remnants, respectively, and prophage 1 is absent from the *S.* Virchow SL491 genome. Both *S.* Virchow strains contain an intact prophage which harbors the virulence gene *sopE* and shares 92–99% nucleotide identity over 67% of the *S.* Typhimurium Gifsy-1 prophage which carries the GogB Type III secreted effector protein (Figure 5). SopE is a Type III secreted effector protein that induces membrane ruffling and promotes bacterial entry into host cells [66–68]. A previous study has revealed that the *sopE* gene and 200 bp of flanking sequences (referred to as the *sopE* cassette) is sporadically distributed on a lambdoid prophage similar to the Gifsy-2 prophage among other *Salmonella* serovars including *S.* Gallinarum, *S.* Dublin and *S.* Enteritidis and on a non-Gifsy prophage in *S.* Typhi [69]. It has been proposed that the *sopE* cassette was transferred between bacteriophage families by homologous recombination [69], a contention that is supported by our observation in *S.* Virchow. Although the SopE prophage in Virchow is significantly different to the other known SopE prophages in other *Salmonella* genomes, the 1.2 kb SopE cassette is 97% identical to the cassettes in *S.* Typhi CT18 and 93% identical to the ones found in *S.* Gallinarum, *S.* Dublin and *S.* Enteritidis. PCR amplification of the *sopE* gene and across the boundaries of the *sopE* cassette was used to confirm the presence of this SopE prophage in SVQ1, SL491 and the other 45 *S.* Virchow isolates (Additional file 6: Table S6). The prevalence of the SopE prophage in the *S.* Virchow SL491 genome and in all Australian isolates tested suggests that it is a defining feature of *S.* Virchow.

## Discussion

*S.* Virchow is of significant public health importance and has a high prevalence in Australia, Asia and Europe [6, 70]. Isolates within this serovar show high levels of genetic relatedness that make discriminating between strains in outbreak investigations difficult [14]. The *S.* Virchow SVQ1 genome has provided the opportunity to investigate potential targets for sub-typing closely related *S.* Virchow isolates. Based on our comparisons of the genomes of *S.* Virchow SVQ1 and *S.* Virchow SL491 we identified 178 genes that contain SNP or small indel differences between these strains. However, when testing a subset of these candidate targets using a collection of 45 *S.* Virchow isolates collected from different outbreaks within Australia only one out of the 11 genes tested could discriminate SVQ1 from all other *S.* Virchow strains in this study. The gene is predicted to encode a pyruvate-flavodoxin oxidoreductase, a conserved housekeeping gene in *Salmonella*, which we propose could be used as part of a typing scheme to discriminate between *S.* Virchow PT8 isolates. The lack of other discriminatory SNPs in the set of genes examined is likely due to the selection bias introduced by comparing only two genome sequences. Given the growing availability of high-throughput sequencing technologies we expect that further discriminatory SNPs will be identified by WGS of multiple *S.* Virchow isolates rather than through PCR-based validation of the remaining candidate genes identified in this study. Furthermore, although routine pathogen surveillance and outbreak investigation will increasingly be carried out by WGS [71], there remains a need for simple molecular genotyping tests.

In this study we found that the CRISPR-1 region could be used to discriminate *S.* Virchow PT8 isolates. CRISPRs were selected as a genotyping target because they were found to be one of the most rapidly evolving regions in bacterial genomes [72]. CRISPR typing has also been successfully applied to more than nine other *Salmonella* serovars [32, 33]. Despite sharing nearly all CRISPR-1 spacer sequences in common, three allelic types of CRISPR-1 were observed in the Australian strains including one associated with PT17 strains. However, the different alleles of CRISPR-1 are caused by deletions of single spacers rather than the acquisition of new spacers. In contrast, CRISPR-1 from S. Virchow SL491 contains 34 spacers not found in Australian isolates suggesting evolutionary differences. Even though CRISPRs are considered to be rapidly evolving elements both CRISPR-1 and CRISPR-2 in the Australian S. Virchow strains have not accumulated new spacer sequences over a seven year period, suggesting that the US strain S. Virchow SL491 has been exposed to a greater variety of mobile DNA.

There is sufficient variation within CRISPR-1 that it can be used to discriminate between closely related *S.* Virchow strains. Despite their repetitive nature, CRISPRs can also be compared using WGS data, as the repeat units in the CRISPRs are short (only about 30 bp long) and are separated by variable, non-repetitive spacer sequences. Most currently available sequencers can produce reads long enough to span these repeat units and overlap with the spacers allowing for correct assembly, as has been recently demonstrated in a study of 102 newly sequenced *S. enterica* genomes [38, 47]. Due to relatively large size of the CRISPR-1, using Sanger sequencing to sequence the whole region is time-consuming, however, we have observed three allelic types of CRISPR-1 in local *S*. Virchow strains that differ by the deletion of a single spacer sequence. Therefore, it a PCR based assay to determine the presence/absence of a specific spacer sequence by designing primers that bind to conserved spacers that flanks a deletion site.

Genomic comparisons between *S.* Virchow SVQ1 and *S.* Virchow SL491 revealed that lateral gene transfer is the major contributor for variation in the chromosome, as for other enteric bacteria. Excluding plasmids, 0.2% of SVQ1 genome is not shared with SL491. Conversely, 4.1% of the SL491 genome is not present in SVQ1. The bulk of the non-shared DNA in SL491 is associated with prophage regions, which are absent in SVQ1. This is a common theme in *Salmonella,* as prophages are known to contribute significantly to variation in strains of the same serovar [73]. Comparisons with other published *Salmonella* genomes revealed several regions of difference in *S.* Virchow genomes, including genomic islands located within regions in the chromosome that are common DNA integration sites in other serovars. For example, tRNA-*leuX* is a region of the chromosome that is often associated with foreign DNA in other *Salmonella* and *E. coli* strains [74]. In *S.* Virchow, the GI-*leuX* appears to encode the remnants of an integrative conjugative element that is distinct from other well-characterized ICE representatives, including the *Salmonella* SPI-7 family [75]. Although the degradation of the GI-T4SS conjugal transfer region indicates that the *S.* Virchow GI-*leuX* is no longer self-transmissible, the island is present in all 47 *S.* Virchow isolates tested in this study suggesting that there may be a selective advantage to retaining one or more of the encoded cargo genes.

*Salmonella* employs the SPI-1 Type III secretion system to translocate effector proteins into host cells [76]. These effectors then manipulate host cellular function to enhance the invasiveness and survival of *Salmonella*. SopE is an effector that is responsible for entry into epithelial cells by inducing cytoskeleton rearrangement and membrane ruffling causing the membrane of the cell to wrap around and engulf the bacterium, a process called macropinocytosis [66, 68, 77]. Knocking out the SopE effector in *S.* Dublin prevented invasion and attenuated disease [78]. Thus, it is believed that the acquisition of the *sopE* gene was an important step in the emergence of epidemic *Salmonella* serovars [79, 80]. Here we report that *S.* Virchow encodes SopE on a Gifsy-1-like prophage that is dissimilar to the SopE prophages found in other *Salmonell*a serovars. The presence of this virulence factor in different bacteriophages might increase the efficiency of horizontal transfer of *sopE* between different strains by increasing the host range and helping to evade immunity imposed by other resident prophages and CRISPRs [69].

## Conclusions

We have undertaken a comparative analysis of the *S.* Virchow SVQ1 genome and identified several genomic islands, prophages and other regions of difference that are characteristic of *S.* Virchow. We have demonstrated that Sesv_1374 and the CRISPR-1 region are genotyping targets that can discriminate between closely related *S.* Virchow isolates of the same phage type. The genotyping targets described in this study could be used in conjunction with other *Salmonella* genotyping targets to provide enhanced resolution of *S*. Virchow strains involved in different outbreaks. Additional genome sequencing of *S.* Virchow strains will help to evaluate the effectiveness of CRISPR typing for outbreak investigations and identify other potential genotyping targets. Considering *S.* Virchow’s public health importance as a human pathogen, the availability of the *S.* Virchow SVQ1 genome is a vital step for understanding the evolution and global distribution of this serovar and the mechanisms in which it causes invasive infections.

## Methods

### Bacterial strains

The strain sequenced in this study, *S.* Virchow strain SVQ1 (phage type 8) is a clinical isolate obtained in 2007. The strain was isolated from an outbreak in Queensland, Australia [9]. *S.* Virchow SL491, for which a genome sequence is available [GenBank:ABFH00000000.2], was included in this study and was phage typed by the Microbiological Diagnostic Unit (MDU), Victoria. *S.* Virchow SL491 was isolated in 2005 from a patient in the USA, however, prior to onset of illness the patient had visited India [38]. A total of 45 other *S.* Virchow isolates belonging to four phage types (PT8, PT17, PT25 and PT34) from various locations in Australia were also in this study (see Table 1).

### Whole genome sequencing, assembly and annotation

The genome of *S.* Virchow SVQ1 (PT8) was sequenced using Roche 454 GS-FLX (Australian Genome Research Facility, Brisbane, Australia) producing 340,790 single-end shotgun reads of an average length of 240 bp. The genome was assembled using 454/Roche gsAssembler 2.3.1 (Newbler) into 54 contigs between 293 bp and 432,538 bp in length (N50 contig size, 205,097 bp) with an average 17-fold read coverage depth. Contig scaffolds were built and ordered based on an optical map (Opgen Inc, Gaitherburg MD, 20878) that was generated for the genome [81, 82]. The optical map was also used to check for misassemblies or genome rearrangements and to confirm contig order. Consed [83] was used to check the underlying reads to determine any collapsed repeats that separate adjacent contigs. This approach allowed us to assemble 46 of the 54 contigs into 10 scaffolds that were ordered according to the *S.* Virchow SL491 genome. The remaining eight unscaffolded contigs corresponded to four plasmids and collapsed repeat contigs that encode rRNA operons, respectively. BLAST comparison of all *S.* Virchow SVQ1 contigs with *S.* Virchow SL491 identified scaffold gaps corresponding to each of the 7 rRNA operons in *S.* Virchow SL491. Examination of paired-end read location from edge of each contig gap suggested that like *S.* Virchow SL491, *S.* Virchow SVQ1 encodes 7 rRNA operons. The draft genome was automatically annotated using SUGAR (Simple Unfinished Genome Annotation Resource) as previously described [84]. Automatic annotation was carried out using BLASTp [49] in a hierarchical approach that prioritised a high-quality manually curated annotations by using a diminishing BLASTp identity thresholds against databases comprising proteins from i) *Salmonella* Typhi str. CT18 genome [GenBank:AL513382] [41], ii) all *Salmonella* genomes iii) swiss-prot or iv) uniprot. tRNA genes were predicted using TE-SCAN [85]. Subsequent manual annotation of genomic islands, prophage and CRISPR sequences was carried out using Artemis [86] and the results of Pfam [87], TIGRfam [88] and COGs [89] searches. Prophages were also characterized using the PHAST phage annotation server [90]. This Whole Genome Shotgun project has been deposited at DDBJ/EMBL/GenBank under the accession [GenBank:AZMP00000000] (Bioproject: PRJNA178788). The version described in this paper is version AZMP01000000.

### Variant prediction

The draft genome of *S.* Virchow SVQ1 (PT8) was compared to the previously published draft genome of *S.* Virchow SL491 (PT25) to identify genes with at least one single nucleotide polymorphism (SNP) that may be suitable genotyping markers. The MUMmer package [91] was used to align the contigs from the genome of *S.* Virchow SVQ1 to the genome sequence of *S.* Virchow SL491 and identify indel and SNP variants. This approach was also used to predict SNPs between *S.* Virchow SVQ1 and *S.* Heidelberg SL497 [GenBank:CP001120] [92]. A custom Perl script was used to remove any SNPs inside or flanking homopolymer tracts of longer than four nucleotides, as errors in base calling can occur at homopolymeric tracts with 454 sequencing [93]. A final filter step removed SNPs with a read coverage of less than five reads or which were located within 10 nucleotides of contig ends.

### PCR amplification and sequencing

Polymerase Chain Reaction (PCR) was used to amplify 11 genes predicted to contain SNPs in 47 *S.* Virchow isolates including the sequenced strains, *S.* Virchow SVQ1 and *S.* Virchow SL491. PCR was used also used to validate the presence of the SopE prophage and selected genomic islands in local *S.* Virchow isolates by amplifying regions within each island and the boundaries at both ends. The CRISPR-1 region was also amplified from 15 strains and were sequenced both forward and reverse using Big Dye V3.1 Sequencing Kits (Applied Biosystems, Life Technologies) and analyzed on the ABI 3130 Sequencer (Applied Biosystems, Life Technologies, Australia). The primers for amplifying CRISPR-1 were designed to bind to the location 5′ and 3′ outside of the CRISPR loci and to conserved spacers between the two *S.* Virchow genomes. Primers used in this study are listed in Table 4.

**Table 4:** **List of primers used to validate genotyping target and genomic features in** ***S.*** **Virchow**

Name	Primer sequence	Length (bp)	Direction	Product size (bp)	Target
SopE-A/F	GAGTCGGCATAGCACACTCA	20	Forward	474	SopE (Sesv_0764)
SopE-A/R	CAACACACTTTCACCGAGGA	20	Reverse		
SopE-B/F	GGCGTGGGAAAGTTTCAGTA	20	Forward	1328	SopE cassette (3′ region)
SopE-B/R	ATGACGTTTTTACGCCAAGC	20	Reverse		
SopE-C/F	CGGGGTCTTTACTCGCACTA	20	Forward	923	SopE cassette (5′ region)
SopE-C/R	CACTCAACCACCACAACAGG	20	Reverse		
leuX-A/F	TTAAATGTGGCGAACAGCAG	20	Forward	2239	GI-leuX (internal)
leuX-A/R	AGTGCCCGGAAAGAAACTCT	20	Reverse		
leuX-B/F	CGGACGCCATATCCATATTC	20	Forward	1120	GI-leuX (5′ boundary)
leuX-B/R	CCTGAATACTGGTCGGGAAA	20	Reverse		
leuX-C/F	GTAGATTGGCAACCGAAAGG	20	Forward	876	GI-leuX (3′ boundary)
leuX-C/R	GAGATGAAACGTTCGTGCAA	20	Reverse		
pheV-A/F	GCGGCAAGGTAAAATGTGTT	20	Forward	1687	GI-pheV (internal)
pheV-A/R	GGTGATTTACGTGCGGTCTT	20	Reverse		
pheV-B/F	TTCTGCTGGTGATGAAGTGC	20	Forward	1138	GI-pheV (5′ boundary)
pheV-B/R	TCCAGATATGGGCTTTCAGG	20	Reverse		
pheV-C/F	GATAGTTTCCGCCACCTGAA	20	Forward	1337	GI-pheV (3′ boundary)
pheV-C/R	GAGAGAACTGGAGCCACAGG	20	Reverse		
SV-0065-F	GCAGAAAGCCTGTCAGGAAC	20	Forward	856	Sesv_0065
SV-0065-R	CACCGGGTTAAAAGGGATCT	20	Reverse		
SV-1374-F	TTTTACGGTCTGGGAAGCGAC	21	Forward	623	Sesv_1374
SV-1374-R	TATGCGGATTAACCGCCTGC	20	Reverse		
SV-0106-F	GGGCCTGCATTTCTTGTCTA	20	Forward	935	Sesv_0106
SV-0106-R	GCCCTTTCTGGATAAGACGA	20	Reverse		
SV-0279-F	CGCAGGTACGCGTGTTATTA	20	Forward	814	Sesv_0279
SV-0279-R	CCGTCGGTGATATTTTCCAC	20	Reverse		
SV-0317-F	GCGCTTAGTCGGCTATTGAC	20	Forward	805	Sesv_0317
SV-0317-R	TACAACCGAATTCACGGACA	20	Reverse		
SV-1243-F	GTTTTGCTGGTTTGGCATTTG	21	Forward	742	Sesv_1243
SV-1243-R	GTCGAACGAACCCAGTCCATG	21	Reverse		
SV-1046-F	GTATGGCGGCAATCATCGTTG	21	Forward	804	Sesv_1046
SV-1046-R	CCTCCTTGAGGACAGCCAACG	21	Reverse		
SV-1509-F	CCAACCGCCTGTACACTTCT	20	Forward	720	Sesv_1509
SV-1509-R	TCGCAGACAACGACTTCATC	20	Reverse		
SV-0512-F	GAAGGTGTACCCGCCAGATA	20	Forward	714	Sesv_0512
SV-0512-R	GGTGGTAACGCTGATGGACT	20	Reverse		
SV-1129-F	CGTTGTTAAATGCGTGGTTG	20	Forward	987	Sesv_1129
SV-1129-R	GGCTGGTAACGACTGGAAAA	20	Reverse		
SV-0619-F	TTTCACCGATGAACCCGTGAC	21	Forward	760	Sesv_0619
SV-0619-R	CGACGGATATGATCGCTCCAG	21	Reverse		
C1-F1	GATGTAGTGCGGATAATGCT	20	Forward	1405	CRISPR-1
C1-R1	CTCATCTCCCCAGATTTTTG	20	Reverse		
C1-F2	CGTAACGTTTAAGCGTGGAAAG	22	Forward	399	CRISPR-1
C1-R2	CGCTTACGATACAATGATGGTC	22	Reverse		
C1-F3	CAGTCACAATCTTTTGCGGC	20	Forward	1497	CRISPR-1
C1-R3	GTTTCTTTTCTTCCTGTTG	19	Reverse		
C1-F4	TCCCACTTATCAAATTTAGCC	21	Forward	578	CRISPR-1
C1-R4	GCCATCGTAGCGGATTTCAGA	21	Reverse		

### Bioinformatics analysis

Pairwise whole genome comparisons of *S.* Virchow SVQ1 with 27 *Salmonella* genomes (Table 3) were performed using BLASTn and visualized using the Artemis Comparison Tool [94]. Circular visualization figures were made using BRIG (BLAST Ring Image Generator) [59] and linear visualization figures were made using Easyfig [50]. CRISPR amplicon sequences were assembled using CLC Genomic Workbench (http://www.clcbio.com/). Similarity searches of the non-redundant nucleotide database and whole-genome shotgun contigs were carried out using the NCBI BLAST portal. The absence in *S.* Virchow SVQ1 of genomic regions present in *S.* Virchow SL491 was confirmed by mapping the 454 reads against the *S*. Virchow SL491 genome as a reference. Prior to mapping, the quality of the 454 reads was checked with FastQC (http://www.bioinformatics.babraham.ac.uk/projects/fastqc/). Reads that were shorter than 200 bp were removed and the remaining reads were trimmed by 10 nucleotide from the 5′ end and 30 nucleotide from the 3 end using PrinSeq-Lite [56]. Read mapping was performed using BWA-SW (Smith Waterman) [57] with default parameters.

## Electronic supplementary material

Additional file 1: Table S1: List of plasmids in the genome of S. Virchow SVQ1. (XLSX 37 KB)

Additional file 2: Table S2: List of protein coding sequences that contain at least one SNP or indel between SVQ1 and SL491. (XLSX 52 KB)

Additional file 3: Table S3: List of Single nucloetide polymorphisms test. (XLSX 41 KB)

Additional file 4: Table S4: Salmonella Pathogenicity Island in S. Virchow SVQ1. (XLSX 39 KB)

Additional file 5: Table S5: Regions of differences identified in S. Virchow SVQ1. (XLSX 50 KB)

Additional file 6: Table S6: PCR results of S. Virchow unique regions. (XLSX 52 KB)
